# Grade-Level Differences in the Profiles of Substance Use and Behavioral Health Problems: A Multi-Group Latent Class Analysis

**DOI:** 10.3390/ijerph21091196

**Published:** 2024-09-10

**Authors:** Kechna Cadet, Ashley V. Hill, Tamika D. Gilreath, Renee M. Johnson

**Affiliations:** 1 Department of Epidemiology, Columbia University Mailman School of Public Health, New Yok, NY 10032, USA; kc3010@cumc.columbia.edu; 2 Division of Community Health Sciences, University of Illinois Chicago School of Public Health, Chicago, IL 60612, USA; ahill68@uic.edu; 3 Center for Health Equity and Evaluation Research, Texas A&M University, College Station, TX 77843, USA; 4 Department of Health Behavior, School of Public Health, Texas A&M University, College Station, TX 77843, USA; 5 Department of Mental Health, Johns Hopkins Bloomberg School of Public Health, Baltimore, MD 21205, USA

**Keywords:** substance use, adolescent, health risk behaviors

## Abstract

We investigated associations between polysubstance use and behavioral problems among adolescents. Because substance use becomes more developmentally normative with age, we examined whether polysubstance use was less likely to co-occur with behavioral problems among older (vs. younger) adolescents. Using data from a nationally representative survey of US high school students, we compared the association between polysubstance use (i.e., use of alcohol, cannabis, tobacco/nicotine, and illicit drugs) and behavioral problems (i.e., suicide attempts, depressive symptoms, poor school performance, and sexual risk behaviors) by grade level. We conducted latent class analysis (LCA) to characterize patterns of polysubstance use, and multi-group LCA to estimate invariance by grade. Among the three latent classes that emerged, classes were distinguished by having low, moderate, and high probabilities for behavior problems and use of substances. Class I comprised 52% of the sample, whereas classes II and III comprised 35% and 12% of the sample, respectively. The multi-group LCA showed that younger adolescents had a higher relative probability of co-occurring problem behaviors and polysubstance use. Findings may be helpful in targeting screening and prevention efforts of high school students by grade. Specifically, our results provide evidence that associations between behavioral problems and alcohol/drug use are weaker in later high school grades, suggesting that substance use may not be a weaker marker of behavioral problems for students in higher grades.

## 1. Introduction

Alcohol, cannabis, and cigarettes are the most commonly used substances among 12- to 17-year-old adolescents, with annual prevalence rates of 24%, 13%, and 13%, respectively [1,2]. The high school years in particular are marked by widespread initiation of substance use, where prevalence of use increases with each additional grade [3]. For example, the lifetime prevalence of cannabis in 2022 was 24% in 10th grade and 38% in 12th grade, based on data from the Monitoring the Future study [4]. Additionally, an estimated 60% of US adolescents reported initiating use of illicit drugs by the age of 14 [5]. Substance use in adolescence is well established as a risk factor for later substance use disorders (SUDs), with use earlier in adolescence representing higher risk [6,7]. Historically, there has been separate research on use of different substances by adolescents. However, recent literature has shown that substance use often occurs concurrently in the form of polysubstance use [8]. The term polysubstance use is a vague term describing the use of at least two psychoactive substances, either simultaneously (in the same instance), concurrently (within a defined period of time), or in combination (mixing two substances together) for therapeutic or recreational purposes [9]. The wide-ranging complexity of polysubstance use behavior posed challenges with regard to assessment, until recent innovative statistical methods such as the person-centered approach of latent class analysis emerged [9]. Two recent systematic reviews summarizing latent classes of polysubstance use in adolescents found the most common polysubstance use class constituted alcohol, cigarettes, and marijuana with and without other drugs [5,6]. This indicates that polysubstance use may be a developmentally normative behavior, not an exception. Identifying specific substance use profiles and how they shift across grade levels will help clarify prevention needs and enhance intervention programming.

Unfortunately, much of the literature on adolescent polysubstance use has constituted only a few substances. Furthermore, polysubstance use is associated with higher risk of negative behavioral outcomes than the use of a single substance, for example, adolescents who engage in polysubstance use are more likely to fail at school when compared to single users or non-users [5,10]. Because patterns of drug use change rapidly over the 4-year course of high school, we investigate grade-level differences in associations between substance use and emotional and behavioral problems among US high school students. In latent class analysis (LCA) studies, youth have been classified as ‘mostly abstainers’, ‘alcohol experimenters’, and occasional and frequent ‘polysubstance users’, with abstainers being the largest group and high polysubstance use being the lowest [11]. Those in the polysubstance use groups had high levels of past 30-day use of frequent alcohol, tobacco, and marijuana [11]. In LCA studies of polysubstance use, as the school grade increased, the risk of the occasional and frequent polysubstance use class also increased in comparison to the mostly abstainers’ class [11].

More recently, the literature suggests that polysubstance use varies in the extent and profile of use across adolescents, and poor school performance and negative behaviors may be more related to certain substance use profiles than others [10]. Problem behavior theory suggests that adolescent risk behaviors are interrelated, indicating a syndrome of problematic behavior; it further suggests that multiple risk behaviors in adolescence complicate a successful transition to emerging adulthood [12,13]. Problem behavior theory posits that the early initiation and developmental transitions in risk behaviors such as drug use are related to are negative distal psychosocial consequences [14,15]. There is strong evidence for an association between emotional and behavioral problems with polysubstance use. Individual-level indicators include the following: (1) any use in adolescence, (2) use of multiple drugs, (3) use that occurs within the context of a constellation of behavioral health problems, such as depression, sexual risk behaviors, or violence. Evidence shows that by the time an individual reaches adolescence, adverse childhood events may have occurred, and they may be engaging in sexual risk behaviors, report negative emotional states, and lower levels of family support, all of which are associated with higher rates of substance use within this developmental period [16,17,18]. These adverse psychosocial experiences may predispose adolescents to increased risk behaviors such as early substance use initiation, continual use, and negative substance-related consequences [16]. There are many reasons why adolescents engage in substance use, including the desire to have novel experiences, attempts to cope with emotional problems, availability of drugs within their environment, or peer pressure [19].

Adolescence is a critical developmental period involving significant psychological, physical, emotional, and socio-behavioral changes [20]. Therefore, preventing or delaying initiation, as well as interventions to address use, are key targets for school-based health programs. Although adolescent substance use is a marker of risk for a later substance use disorder, most high school students that engage in substance use will not develop a disorder. The high prevalence of substance use among adolescents makes it difficult to determine which patterns of use are likely to increase risk for SUDs and other problems related to substance use. Thus, the challenge for prevention relates to the application of surveillance data to predict patterns of adolescent substance use that may demonstrate risk for SUD and related problems.

Historically, polysubstance use was difficult to operationalize due to the heterogeneity of drug use configurations, with many studies using a variable-centered approach that focuses on the relationships among variables and assumes homogeneity across the polysubstance using population in regards to how the behavior is reported and how predictors influence relationships among variables. They assess the average influence of predictors on the mean of a particular sample [21,22,23]. However, the emergence of latent class analysis (LCA) in recent years is a common strategy for identifying salient categories attributable to polysubstance patterns [24]. Taking a person-centered approach, LCA is a subtype of nonparametric cluster analysis that is often used to identify classes of individuals who share similar phenotypic profiles, such as drug use [22,25]. LCA enables the identification of groups that are homogenous concerning their drug use classes. An advantage of the technique is that individuals are assigned to classes based on a probabilistic basis, therefore allowing comparison of polysubstance use rates across classes [25]. A large number of recent studies have used LCA to reveal classes of homogenous illicit drug use phenotypes [8,26,27,28,29]. Given the heterogenous nature of adolescent substance use [7,30], LCA has become a popular approach to identify salient typologies among this population [5,6,31]. Our study uses LCA to enhance our understanding of variation in profiles of polysubstance use in relation to behavioral problems. This approach is built on the scientific premise that adolescents often use multiple drugs of abuse, and that latent variable statistical methods can aid in identifying the underlying patterns of use [5,6,8].

The aim of this study is to explore how the association between polysubstance use and emotional/behavioral problems changes as adolescents progress through grade levels (9th to 12th) using data from a national sample of US high school students. We will also employ a multi-group LCA approach to explore measurement invariance by grade levels. One advantage in using the multi-group LCA approach is that it does not constrain profiles to be the same across different demographic characteristics; rather we will explore the extent to which profiles of polysubstance use vary across grade in terms of the type and size of each latent class, but also in the specific item functions within each latent class [32,33]. This analytical tool has been implemented previously to determine whether class membership and structure differ according to important sample characteristics such as gender or race/ethnicity [31,34,35,36,37]. Distinguishing specific patterns of polysubstance use that signify increased risk for adverse outcomes in emerging adulthood will enable us to offer practitioners a new way to use adolescent surveillance data to monitor unhealthy patterns of substance use, to inform their efforts at policy development, and to support establishing targeted services and preventive interventions. Our approach is based on the literature demonstrating that the following three groups of use are high risk: (1) adolescents who engage in substance use at younger ages or before their same-grade peers; (2) adolescents who use a greater number of substances, who use substances more frequently, or who use substances in larger amounts; and (3) adolescents whose substance use occurs within the context of other emotional and behavioral health problems.

## 2. Materials and Methods

### 2.1. Study Sample

The data are from the 2017 National Youth Risk Behavior Survey (YRBS), a school-based survey of high school students on health and behaviors. Detailed information about the methodology of YRBS has been described elsewhere [38]. Survey administration is coordinated through the 45 participating states. Sampling for each participating state involves a two-stage stratified clustered sampling design, with schools sampled and then classrooms sampled within the selected schools. The survey was administered using a machine-scannable paper booklet and a pencil during class. The data for each state are weighted to represent the state’s public high school population. States failing to meet a response rate criterion of >60% are not included in the National YRBS dataset. The YRBS includes several ethical considerations to ensure the data are anonymized and the personal identifiable information is removed. Additionally, parental consent and youth assent are obtained during the data collection phase.

### 2.2. Measurement of Study Variables

Students reported their sex (male/female), grade (9th–12th), race, and ethnicity. Responses to the latter were recoded as follows: (1) monoracial, non-Hispanic White; (2) monoracial, non-Hispanic Black; (3) monoracial, Non-Hispanic Asian; (4) Hispanic/Latino, any race; (5) American Indian, Alaska Native, Native Hawaiian, and Pacific Islander; and (6) all other, which is primarily comprised of bi- and multi-racial students.

Substance use variables were based on responses to items about frequency of lifetime and/or past 30-day use of alcohol, cannabis, combustible tobacco (i.e., cigarettes, cigars, and cigarillos), electronic cigarettes (e-cigarettes), prescription opioids (non-medical use only), cocaine, heroin, and methamphetamine. For alcohol, cannabis, combustible tobacco, and e-cigarettes, we recoded the responses as follows: no lifetime use; lifetime use but no past 30-day use; past 30-day use on <6 days or <6 times; and past 30-day use on >6 days or >6 times. Additional variables included the following: any past 30-day binge drinking (i.e., 4–5 drinks on one occasion), any lifetime illicit drug use (coded as yes for students who reported use of cocaine, heroin, or methamphetamine), and any lifetime non-medical prescription opioid use.

All 5 variables representing emotional or behavioral problems were binary. Emotional distress indicated students’ endorsement of “feeling sad or hopeless every day for two weeks or more”; poor school performance was indicated by students describing their average grades in school as mostly Cs, Ds, or Fs; and number of lifetime sexual partners was classified as ≥3 versus 2 or fewer. Additional variables were any past 12-month suicide attempts and having had more than one physical fight in the past 12 months.

### 2.3. Statistical Analysis

Latent class analysis (LCA) was used to identify profiles of substance use and behavioral problems using the study variables described above as indicators. First, we tested whether endorsement of items was similar for respondents in each grade (i.e., assessed measurement invariance) to determine the appropriate analytic strategy. Because the test indicated invariance by grade (*p* < 0.001), we then conducted multi-group LCA (MG-LCA) with grade as the grouping variable. Although separate LCAs across grade levels can provide some insights, a multi-group LCA offers a robust, consistent, and comprehensive understanding of the latent class structures and allows for direct statistical comparisons across the different grades. The best-fitting MG-LCA model was selected from standard fit criteria, i.e., the G^2^ likelihood ratio chi-square value, Bayesian Information Criterion (BIC), Akaike Information Criterion (AIC), adjusted values of the AIC, and entropy. Lower values indicate optimal model fit. We present conditional probabilities of individuals within latent classes responding to each item, and latent classes were interpreted based on variables that have the highest reported conditional probabilities across classes. The most appropriate model was selected based on the fit statistics and substantive theory. We used multinomial logistic regression to estimate the odds of class membership by race/ethnicity, grade, and sex. Sample selection and survey non-response were accounted for by using sample weights, and stratum variables were accounted for by the complex survey design.

## 3. Results

The weighted proportion of students in ninth, tenth, eleventh, and twelfth grades were 23.7%, 25.7%, 23.9%, and 23.1%, respectively (*n =* 14,765). The subsamples for each grade were similar with respect to the percentages of students by sex and race/ethnicity (Table 1). For students in each grade level, the lifetime prevalence of illicit drug use (i.e., cocaine, methamphetamine, heroin) was <10%, whereas the lifetime prevalence of non-medical prescription opioid use was >10%. Past 30-day substance use was highest for alcohol and cannabis, and lowest for combustible tobacco. Considering the behavioral problems, the prevalence of suicide attempts was the lowest, whereas emotional distress was the highest.

The Rao–Scott chi-square tests indicated that there were statistically significant differences by grade for all of the substance use and behavioral problems variables (*p* < 0.001). The prevalence of use for most substances was higher with each grade level. The difference in the prevalence by grade was largest for alcohol use (19.3% and 42.3% among 9th and 12th graders, respectively) and ≥3 sexual partners (5.7% and 25.5% among 9th and 12th graders, respectively).

### 3.1. LCA of Substance Use and Behavior Problems

We selected a 3-class LCA model based on the fit statistics (Table A1). The conditional probabilities for each indicator are presented in Table 2. The largest proportion of students were in Class I (52.3%), which is characterized by low probabilities for use of all the substances and for most of the behavior problems. Class II comprised 35.3% of the students, and conditional probabilities indicate that students in this class were likely to report lifetime, but not recent use of tobacco/nicotine, and that most reported at least lifetime use of cannabis and alcohol, which was moderately common. Class II conditional probabilities for emotional distress and poor school performance were 0.40 and 0.29, respectively. Class III contains 12.4% of the students and is characterized by conditional probabilities above 0.50 for several indicators of substance use and behavioral problems, including the following: illicit drug use, binge drinking, past 30-day use of alcohol, emotional distress, and three or more sexual partners. Based on the conditional probabilities of each class, we named Class I the ‘low substance and problem behaviors’, Class II is the ‘moderate substance use and problem behaviors’, and Class III is the ‘high substance use and problem behaviors’ to better reflect the characteristics of these typologies.

Odds of membership in the latent classes by sex, race/ethnicity, and grade levels are presented in Table 3. Males had 10% lower odds of being in Class II and 16% greater odds of being in Class III, both relative to Class I. Relative to White students, students in all race/ethnicity groups except Asian were more likely to be in Class II versus Class I. American Indian, Alaska Native, Native Hawaiian, and Pacific Islander students were 50% more likely to be in Class II, followed by all other races (28%), Hispanic/Latino (22%), and Black adolescents (15%). White adolescents were more likely to be members of Class III than Class I, while all other racial groups are less likely to be members of Class III.

We also considered odds of class membership by grade. Relative to 9th graders, students in grades 10–12 were significantly more likely to be in Class II versus Class I. Students in grades 10–12 were also more likely than 9th graders to be in Class III versus Class I, and their odds of class membership for Class III were higher than for Class II.

### 3.2. Measurement Invariance by Grade Level

The magnitude of the association between grade and class was substantial, justifying a test of measurement invariance by grade level. The test for measurement invariance by grade involved estimating the difference between the fit of the restricted (i.e., assumption of no variation by grade) and unrestricted models, with G^2^. The difference was then compared to a χ^2^ distribution with degrees of freedom (df) equal to the differences between the df for the restricted and unrestricted models. The difference was G^2^ = 1280.44 (df = 180), which was statistically significant at *p* < 0.0001 (Table A2). Since the test provided evidence that odds of class membership were not constant by grade, we then conducted a multi-group LCA, with grade as the grouping factor.

### 3.3. Multi-Group LCA of Substance Use and Behavior Problems by Grade Level

We selected a 3-class multi-group LCA model based on established fit statistics, e.g., log-likelihood, G2, AIC, BIG, and entropy (Table A3). The emergent latent classes for each grade were similar to the classes observed for the LCA with all grades combined. Specifically, Class I was characterized by low probabilities for use of all the substances and for most of the behavior problems, and was named ‘low substance uses and problem behaviors’. Class II was characterized by students who were likely to report lifetime, but not recent use of tobacco/nicotine, and most reported at least lifetime use of cannabis and alcohol, which was moderately common and increased across grade levels. Therefore, this class was named the ‘moderate substance uses and problem behaviors’ class. Class III was named the ‘high substance uses and problem behaviors’ and contains students that heavily endorsed several indicators of substance use and behavioral problems (i.e., conditional probabilities were >0.50), including illicit drug use, combustible tobacco, cannabis use, binge drinking, past 30-day use of alcohol, emotional distress, and three or more sexual partners. 

Class I is largest in size, but the proportion in the class decreases from 9th to 12th grade (Table 4, bottom row). By contrast, the proportion in Class II increases with grade, from 27.7 in 9th grade to 40.6% in 12th grade. The proportion in Class III changes modestly across grades, and is the largest for 11th graders (15.5%). 

Conditional probabilities from the multi-group LCA are presented in Table 4. The likelihood of engagement in specific behaviors among youth in the latent class marked by high engagement in risk behaviors (Class III) varies by grade. Profiles of risk behavior change over adolescence. There are noticeable differences in the risk profiles by grade level; for example, those in Class III use more drugs. For Class III, conditional probabilities for all indicators of substance use were successively higher for each additional grade level. For example, the conditional probabilities for cannabis use on 6 or more days in the past 30 days was 0.38 for 9th graders and 0.64 for 12th graders. The endorsement of three or more sexual partners in Class III increased across grade level (0.34–0.75); however, physical fighting was less likely to be a core feature of Class III in 12th grade. Additionally, emotional distress, poor school performance, and (to a lesser extent) suicide attempts remained stable across grade levels. The 9th graders (*n* = 399) in Class III had the highest probability of reporting a suicide attempt (31%), poor school performance (45%) and physical fighting (51%) compared to 10th, 11th and 12th graders in the high use classes.

## 4. Discussion

This study sought to examine the substance use and emotional/behavioral problem typologies as adolescents progress through 9th to 12th grade using a national sample of high school students in the United States. Identifying the typologies of complex interacting substance use and externalizing symptomologies and behaviors, such as poor school performance, suicide attempts, and emotional distress, can aid in the development of targeted prevention initiatives that meet the needs of this particular subgroup of adolescents.

We identified three classes of substance use and problem behaviors in our high school sample: low substance use and problem behaviors (Class I), moderate substance use and problem behaviors (Class II), and high substance use and problem behaviors (Class III). The high substance use and problem behaviors was the smallest class, and was more likely to be White and male. Similarly, older high school students were more likely than their younger counterparts to be in Class III than Class I, and experienced greater problem behaviors. A 2016 study using the Monitoring the Future data found similar drug use patterns across adolescent 8th, 10th, and 12th grade students, with three patterns of polysubstance use emerging among 12th graders: (1) low substance use (76%); (2) simultaneous use of e-cigarettes and high endorsement of combustible cigarettes, cannabis, illicit drug use, and problematic alcohol use (16%); and (3) e-cigarette use with low level of other illicit drug use (8%) [39]. Polysubstance use in adolescence is a known risk factor for substance misuse in adulthood [40]. Our study illustrates the contemporary patterns of substance use and their correspondingly complex interaction with problem behaviors, which have implications for preventive work among adolescents.

Similarly, within the high substance use and problem behavior class, adolescents endorsed heavy use (using substance six times or more in the past 30 days) of alcohol, cannabis, e-cigarettes, binge drinking, and combustible cigarettes ranging from 26% to 77%. Furthermore, the high substance use and problem behavior class had the highest probability of experiencing suicide attempts, poor school performance, emotional distress, multiple sexual partners, and physical fights. This study provides further evidence for the joint occurrence of polysubstance use and emotional/behavioral problems among adolescents and their increasing rate across grade levels. This can be partly explained by problem behavior theory, which suggests that adolescent risk behaviors are interrelated, indicating a syndrome of problematic behavior; it further suggests that multiple risk behaviors in adolescence complicate a successful transition to emerging adulthood [14,41]. There is strong evidence for an association between emotional and behavioral problems with polysubstance use, although knowledge is limited and disjointed because most studies investigate one-to-one associations, such as associations between use of a specific substance with a few behaviors and emotional risk factors [5,42]. Our approach is an advance over research focused on single behaviors. Findings can inform the practice of health and social services providers who work with adolescents by providing empirical evidence for concurrent screening/monitoring of multiple behavior health indicators as well as tailoring for intervention.

Across grade level, adolescents experienced an increasing probability of endorsing all drug categories. Conversely, many problem behaviors such as poor school performance, suicide attempt, and emotional distress decreased across grade levels. This indicates that drug use becomes more normative throughout high school, and older high school students who engage in substance use may be less likely to have other behavioral problems. Other studies have examined the association between adolescent substance use and academic achievement and risk behaviors [11,43,44]; however, these findings contribute to the extant literature illustrating that adolescents who engage in escalating substance use patterns do not necessarily follow the path of engaging in riskier external behaviors across grade levels.

In addition to identifying heterogenous substance use and problem behaviors among high school adolescents, we demonstrate that these patterns vary by important demographic correlates such sex, race, and grade level. In our study, we found that males were at an increased risk of belonging to the high substance use and problem behavior class compared to their female counterparts. Similarly, the high substance use and problem behavior class was more likely to be White, while all other racial/ethnic minority adolescents (except Asians) were more likely to fall within the moderate substance use and problem behavior class. This aligns with the previous literature that has demonstrated the different typologies of substance use across Black and White adolescents, with Black adolescents demonstrating a lower risk of polysubstance use than White adolescents [31]. Historically, Black adolescents tend to demonstrate a lower prevalence of most substance use than their White counterparts [45]. Despite this, Black adolescents who do engage in substance use are more likely to experience poorer outcomes than their White counterparts [46]; therefore, more research is needed to understand the social contextual factors that lead to these paradoxical differences. Furthermore, in our study, it was unsurprising that the risk of belonging to either the moderate or high substance use and problem behavior class increased linearly with grade level when compared to belonging to the low substance use and problem behavior class. Our findings are in alignment with previous studies that have shown that members of the higher risk polysubstance and problem behavior class tend to be male [8] and are more likely to be White [41,47], with polysubstance use increasing across grade-level [48].

Multi-group analysis revealed that roughly 10–15% of each grade level belonged to the high problem behavior and substance use class. The probabilities of reporting alcohol, cannabis and tobacco use were consistent with prevalence estimates from the 2017 YRBS data. However, of interest was the higher probability of reporting poor school performance, suicide attempts, and fighting in school from 9th graders, in addition to higher rates of substance use. Initiating substance use in early adolescence is linked with continued use and the development of substance use disorders in early adulthood [49,50]. Early substance use initiation may also be indicative of more severe problem behaviors, including self-harm, as found in the higher probability of suicide attempts in 9th graders in the present study. This study provides a more nuanced examination of potential timing for early interventions to reduce substance use that may need to begin prior to the 9th grade, when the constellation of problem behaviors and substance use may be more severe. There is a need for preventive strategies such as implementing mental health support programs and culturally relevant education on substance use and polysubstance use. 

### Limitations/Strengths

Although this study was conducted on a large and nationally representative sample of U.S. adolescents, the present study does have a few limitations. First, the data were cross-sectional in nature, therefore, causal inference cannot be made due to the lack of temporal order regarding problem behaviors and substance use. Future studies should consider utilizing person-centered structural equation modeling within the longitudinal framework. For example, longitudinal latent transition analysis can provide insights on how youths transition across different typologies of concurrent substance use, risk factors associated with the transitions, and help refine or adapt interventions to the changing trends. Furthermore, the adolescent participants were predominantly White and served as a referent group within this analysis; therefore, it is unclear how substance use typologies relate to problem behaviors within racial and ethnic minority groups, such as Black and Hispanic adolescents. To date, there is only one study [31] that has assessed whether patterns of polysubstance use are differentially associated with risk factors and consequences among Black adolescents. Notwithstanding, this is the first study to our knowledge to explore how the association between polysubstance use and emotional/behavioral problems changes as adolescents progress through grade levels. Such information can encourage the development of robust and multi-tiered preventive approaches to suit this population. The findings from this study contributes to the extant literature and enhance our understanding regarding initiation of youth polysubstance use and the related emotional and behavioral factors that can inform targeted interventions to address the unique needs of different demographic groups.

## 5. Conclusions

Altogether, this study provides additional clarity to the relationship and typologies of substance use and problem behaviors among adolescence. Despite the heterogeneity in adolescent polysubstance use, most prevention initiatives and programs target singular use of alcohol or tobacco, with very few programs targeting many substances concurrently [31,51]. To date, there is no evidence-based programming that targets polysubstance use among adolescents. In light of these results, prevention approaches need to take into account the contemporary patterns of polysubstance use across grade levels among high school students.

## Figures and Tables

**Table 1:** Demographic characteristics, substance use, and behavioral problems, by grade.

	9th	10th	11th	12th
Number of students	3921	3715	3602	3383
Sex				
Male	49.9%	49.2%	49.1%	48.7%
Female	50.1%	50.8%	50.9%	51.4%
Race and Ethnicity ^1^				
White	51.4%	53.5%	54.0%	55.6%
Black	14.5%	13.7%	12.6%	12.7%
Hispanic/Latino	10.4%	10.4%	9.4%	8.9%
Asian	2.9%	3.3%	3.9%	4.0%
AI/AN/NH/PI	1.4%	1.0%	1.4%	1.0%
All Other	19.4%	18.1%	18.7%	17.8%
Lifetime Substance Use				
Illicit drug use ^2^	3.5%	4.8%	6.0%	8.1%
Prescription opioid use ^3^	10.7%	12.7%	15.3%	16.9%
Past 30-Day Substance Use				
Combustible tobacco use	5.7%	7.9%	9.9%	14.1%
Binge drinking	7.3%	11.4%	15.4%	21.0%
E-cigarette use	9.4%	11.3%	13.9%	18.3%
Cannabis use	13.1%	18.8%	22.6%	25.9%
Alcohol use	19.3%	27.8%	35.3%	42.3%
Behavioral Problems				
≥3 sexual partners, lifetime	5.7%	11.4%	16.7%	25.5%
Suicide attempt, past 12 months	7.1%	7.2%	5.2%	5.0%
Poor school performance	18.2%	20.3%	22.8%	17.7%
≥2 physical fights, past 12 months	20.8%	19.4%	15.7%	14.0%
Emotional distress, past 2 weeks	29.8%	32.5%	32.5%	31.0%

Sample weights were included in analyses to adjust for survey non-response and sample selection probabilities. ^1^ Students self-reported race; AI = American Indian, AN = Alaska Native, NH = Native Hawaiian, PI = Pacific Islander. The group labeled ‘All Other’ includes students who were bi- or multi-racial, or whose race did not fit into any of the standard categories. ^2^ Includes any use of cocaine, heroin, or methamphetamine. ^3^ Represents non-medical prescription opioid use only.

**Table 2:** Conditional probabilities from LCA 3-class solution for substance use and behavior problems.

	Class I	Class II	Class III
Percentage of sample (*n*)	52.3% (7722)	35.3% (5212)	12.4% (1831)
Illicit drug use, lifetime	0.02	0.17	0.54
Prescription opioid misuse, lifetime	<0.01	0.03	0.34
Combustible tobacco use			
No use, lifetime	0.98	0.53	0.13
No use, past 30 days	0.03	0.40	0.29
Used on <6 days, past 30 days	<0.01	0.04	0.26
Used on ≥6 days, past 30 days	<0.01	0.02	0.32
Binge drinking, past 30 days	<0.01	0.15	0.77
E-cigarette use			
No use, lifetime	0.94	0.35	0.10
No use, past 30 days	0.05	0.51	0.20
Used on <6 days, past 30 days	<0.01	0.10	0.30
Used on ≥6 days, past 30 days	<0.01	0.04	0.40
Cannabis use			
No use, lifetime	0.96	0.36	0.07
No use, past 30 days	0.03	0.34	0.18
Used <6 days, past 30 days	<0.01	0.22	0.30
Used ≥6 days, past 30 days	<0.01	0.07	0.45
Alcohol use			
No use, lifetime	0.74	0.10	<0.01
No use, past 30 days	0.20	0.42	0.03
Used on <6 days, past 30 days	0.06	0.43	0.52
Used on ≥6 days, past 30 days	<0.01	0.04	0.45
≥3 sexual partners, lifetime	0.02	0.19	0.53
Suicide attempt, past 12 months	0.03	0.09	0.20
Poor school performance	0.14	0.29	0.39
≥2 physical fights, past 12 months	0.06	0.16	0.37
Emotional distress, past 2 weeks	0.21	0.40	0.52

Note. Cells with shading have high or very low conditional probabilities: ≤0.05 are shaded in light gray, ≥0.50 are shaded in black. Class I = low substance and problem behaviors; Class II = moderate substance use and problem behaviors; and Class III = high substance use and problem behaviors.

**Table 3:** Odds ratio of membership in latent classes by sex, race/ethnicity, and grade; from LCA 3-class solution for substance use and behavior problems.

	Class I	Class II	Class III
	(*n =* 7722)	(*n =* 5212)	(*n =* 1831)
Sex			
Females	---	---	---
Males	---	0.90 (0.84–0.97)	1.16 (1.04–1.30)
Race/Ethnicity			
White	---	---	---
Black	---	1.15 (1.04–1.26)	0.55 (0.46–0.65)
Hispanic/Latino	---	1.22 (1.08–1.37)	0.68 (0.55–0.83)
Asian	---	0.38 (0.27–0.42)	0.19 (0.12–0.29)
AI/AN/NH/PI	---	1.50 (1.14–1.97)	0.45 (0.39–0.55)
All Other	---	1.28 (1.16–1.41)	1.04 (0.90–1.94)
Grade level			
9th	---	---	---
10th	---	1.52 (1.37–1.68)	1.63 (1.37–1.94)
11th	---	2.02 (1.83–2.23)	2.64 (2.24–3.12)
12th	---	2.55 (2.30–2.83)	3.91 (3.32–4.60)

Note. A triple dash (---) indicates that the group or class served as the reference group. AI = American Indian, AN = Alaska native, NH = Native Hawaiian, PI = Pacific Islander.

**Table 4:** Conditional probabilities from the multi-group LCA 3-class solution for substance use and behavior problems; and probabilities of students within each class, by grade.

	Ninth Graders (*n* = 3921)			Tenth Graders (*n* = 3715)			Eleventh Graders (*n* = 3602)			Twelfth Graders (*n* = 3383)		
Percentage of sample (*n*)	Class I	Class II	Class III	Class I	Class II	Class III	Class I	Class II	Class III	Class I	Class II	Class III
Illicit drug use, lifetime	0.00	0.02	0.28	0.00	0.04	0.35	0.00	0.02	0.33	0.00	0.04	0.52
Prescription opioid misuse, lifetime	0.02	0.16	0.50	0.02	0.17	0.55	0.02	0.15	0.58	0.04	0.18	0.67
Combustible tobacco use												
No use, lifetime	0.98	0.62	0.18	0.98	0.55	0.16	0.97	0.50	0.16	0.94	0.42	0.06
No use, past 30 days	0.02	0.36	0.32	0.02	0.39	0.25	0.03	0.45	0.31	0.05	0.44	0.20
Used on <6 days, past 30 days	0.00	0.02	0.23	0.00	0.04	0.30	0.00	0.04	0.25	0.00	0.08	0.24
Used on ≥6 days, past 30 days	0.00	0.00	0.27	0.00	0.00	0.27	0.00	0.02	0.29	0.00	0.06	0.50
Binge drinking, past 30 days	0.00	0.07	0.63	0.00	0.13	0.78	0.00	0.15	0.73	0.00	0.32	0.81
E-cigarette use												
No use, lifetime	0.96	0.44	0.11	0.96	0.33	0.11	0.93	0.31	0.09	0.91	0.28	0.09
No use, past 30 days	0.04	0.45	0.18	0.04	0.53	0.14	0.07	0.56	0.25	0.09	0.47	0.17
Used on <6 days, past 30 days	0.00	0.08	0.36	0.00	0.10	0.28	0.00	0.08	0.31	0.00	0.16	0.24
Used on ≥6 days, past 30 days	0.00	0.02	0.34	0.00	0.04	0.46	0.00	0.05	0.35	0.00	0.09	0.50
Cannabis use												
No use, lifetime	0.98	0.52	0.08	0.96	0.37	0.08	0.94	0.34	0.07	0.91	0.25	0.02
No use, past 30 days	0.02	0.27	0.21	0.03	0.32	0.15	0.04	0.37	0.19	0.05	0.41	0.08
Used <6 days, past 30 days	0.01	0.17	0.33	0.01	0.24	0.33	0.01	0.24	0.30	0.03	0.22	0.26
Used ≥6 days, past 30 days	0.00	0.04	0.38	0.00	0.07	0.43	0.00	0.05	0.44	0.01	0.12	0.64
Alcohol use												
No use, lifetime	0.82	0.16	0.00	0.74	0.13	0.00	0.71	0.08	0.00	0.62	0.03	0.01
No use, past 30 days	0.15	0.50	0.09	0.21	0.40	0.02	0.22	0.45	0.04	0.28	0.31	0.03
Used on <6 days, past 30 days	0.03	0.33	0.57	0.05	0.43	0.46	0.07	0.42	0.57	0.10	0.55	0.40
Used on ≥6 days, past 30 days	0.00	0.01	0.34	0.00	0.04	0.51	0.00	0.05	0.39	0.00	0.11	0.57
≥3 sexual partners, lifetime	0.01	0.07	0.34	0.02	0.17	0.44	0.02	0.27	0.50	0.07	0.33	0.75
Suicide attempt, past 12 months	0.02	0.14	0.31	0.04	0.11	0.27	0.02	0.07	0.18	0.03	0.06	0.18
Poor school performance	0.13	0.31	0.45	0.14	0.32	0.44	0.17	0.30	0.42	0.12	0.23	0.39
≥2 physical fights, past 12 months	0.06	0.27	0.51	0.06	0.20	0.48	0.04	0.10	0.36	0.03	0.10	0.35
Emotional distress, past 2 weeks	0.19	0.45	0.57	0.23	0.41	0.53	0.21	0.40	0.49	0.21	0.33	0.64
Number within class Percentage	2435	1087	399	2005	1341	369	1717	1328	557	1616	1374	393
	62.1%	27.7%	10.2%	54.0%	36.1%	9.9%	47.7%	36.9%	15.5%	47.8%	40.6%	11.6%

Note. Cells with shading have high or very low conditional probabilities: ≤0.05 are shaded in light gray, ≥0.50 are shaded in black. Class I = low substance and problem behaviors; Class II = moderate substance use and problem behaviors; and Class III = high substance use and problem behaviors.

## Data Availability

National YRBS data can be obtained from the CDC. Instructions for data acquisition are available online, accessed on 2 March 2020: https://www.cdc.gov/healthyyouth/data/yrbs/index.htm.

## Appendix A

**Table A1:** Fit statistics for LCA.

Number of Classes	LL	G^2^	AIC	BIC	CAIC	Adj-BIC	Entropy	DF
1	−98,806.21	48,248.42	48,288.42	48,440.42	48,460.42	48,376.86	1	65,515
2	−85,970.22	22,576.44	22,658.44	22,970.04	23,011.04	22,839.75	0.84	65,494
3	−83,442.75	17,521.51	17,645.51	18,116.71	18,178.71	17,919.68	0.81	65,473
4	−82,332.00	15,300.01	15,466.01	16,096.81	16,179.81	15,833.05	0.80	65,452
5	−81,939.65	14,515.31	14,723.31	15,513.71	15,617.71	15,183.21	0.76	65,431
6	−81,594.06	13,824.13	14,074.13	15,024.13	15,149.13	14,626.90	0.76	65,410

Note. The 3-class solution was selected based on optimal fit criteria. Highlighted row indicates optimal class selection.

**Table A2:** Test for measurement invariance by grade level.

	G^2^	df
Unrestricted (U)	25,710.37	26,1895
Restricted (R)	26,990.81	262,075
U-R	1280.44	180
*P*	<0.0001	

Note. The difference is statistically significant, indicating that item-response probabilities vary significantly by grade in the LCA model. Results provide justification for a multi-group LCA.

**Table A3:** Fit statistics for the multi-group LCA.

Number of Classes	LL	G^2^	AIC	BIC	CAIC	Adj-BIC	Entropy	DF
2	−84,732.54	31,981.43	32,069.43	32,403.40	32,447.40	32,263.57	0.84	262,099
3	−82,237.23	26,990.81	27,126.81	27,642.94	27,710.84	27,426.84	0.81	262,075
4	−84,732.54	31,981.43	32,069.43	32,403.40	32,447.40	32,263.57	0.84	262,099
5	−80,748.53	24,013.40	24,245.40	25,125.86	25,241.86	24,757.22	0.76	262,027

Note. The 3-class solution was selected based on optimal fit criteria. Highlighted row indicates optimal class selection.
